# PREDICTION OF SARCOPENIA USING A MULTIPLE BIOMARKER RISK SCORE OVER TWO-YEAR FOLLOW-UP

**DOI:** 10.1093/geroni/igad104.2773

**Published:** 2023-12-21

**Authors:** Hyung Eun Shin, Miji Kim, Chang Won Won

**Affiliations:** Kyung Hee University, Seoul, Republic of Korea; Kyung Hee University, Seoul, Republic of Korea; Kyung Hee University Hospital, Seoul, Republic of Korea

## Abstract

It is necessary to consider multiple biomarkers simultaneously to detect sarcopenia due to its multifactorial etiology. This study is to compare the AUC of multiple biomarker risk score (MBS) that combines multiple biomarkers with that of individual biomarkers and to examine the association of MBS with incidence of sarcopenia in older adults. A total of 1,021 older adults were selected from the Korean Frailty and Aging Cohort Study. Sarcopenia was defined using the Asian Working Group for Sarcopenia 2019 criteria. The MBS was calculated from eight biomarkers (Growth differentiation factor-15, Cystatin-C, Dehydroepiandrosterone, Hemoglobin, Myostatin, Aspartate aminotransferase/Alanine aminotransferase, Estimated glomerular filtration rate, and Blood urea nitrogen) using ridge regression. Receiver operating characteristic (ROC) analysis was conducted to identify an utility of MBS to discriminate sarcopenia. The association of MBS with incidence of sarcopenia were examined by multivariate logistic regression. MBS has an area under the ROC curve (AUC) of 0.71 with an optimal cutoff of 1.76, which is higher than all other individual biomarkers (all, p< 0.01). In prospective study, continuous MBS was positively associated with incidence of sarcopenia after adjusting for confounders (odds ratio[OR]=1.63; 95% CI=1.23–2.17). Participants with high risk (>1.76 score) had a higher odds of sarcopenia than those with low risk (≤ 1.76) (OR=1.82; 95% CI=1.04–3.19). In conclusion, MBS better discriminated the presence of sarcopenia than did individual biomarkers, which can further predict sarcopenia after 2 years in older adults. Thus, combination of multiple biomarkers into single risk score can be helpful for earlier detection of sarcopenia.

